# Magnetic Lenz lenses improve the limit-of-detection in nuclear magnetic resonance

**DOI:** 10.1371/journal.pone.0182779

**Published:** 2017-08-15

**Authors:** Nils Spengler, Peter T. While, Markus V. Meissner, Ulrike Wallrabe, Jan G. Korvink

**Affiliations:** 1 IMTEK – Department of Microsystems Engineering, University of Freiburg, Freiburg, Germany; 2 Institute of Microstructure Technology (IMT), Karlsruhe Institute of Technology (KIT), Eggenstein-Leopoldshafen, Germany; 3 Department of Radiology and Nuclear Medicine, St. Olav’s University Hospital, Trondheim, Norway; nanyang technological university, SINGAPORE

## Abstract

A high NMR detection sensitivity is indispensable when dealing with mass and volume-limited samples, or whenever a high spatial resolution is required. The use of miniaturised RF coils is a proven way to increase sensitivity, but situations may arise where space restrictions could prevent the use of a small resonant coil, e.g., in the interior of the smallest practicable micro-coils. We present the use of magnetic lenses, denoted as Lenz lenses due to their working principle, to focus the magnetic flux of an RF coil into a smaller volume and thereby locally enhance the sensitivity of the NMR experiment—at the expense of the total sensitive volume. Besides focusing, such lenses facilitate re-guiding or re-shaping of magnetic fields much like optical lenses do with light beams. For the first time we experimentally demonstrate the use of Lenz lenses in magnetic resonance and provide a compact mathematical description of the working principle. Through simulations we show that optimal arrangements can be found.

## Introduction

Both nuclear magnetic resonance (NMR) spectroscopy and magnetic resonance imaging (MRI) suffer from an inherently low sensitivity. The signal strength is primarily determined by the equilibrium Boltzmann distribution, with energy levels of the spin states just slightly above the thermal energy. Consequently, the limit of detection (LOD) is up to ten orders of magnitude worse compared to other analytical techniques [1, 2]. This fact severely limits the lowest detectable quantity in NMR spectroscopy and the highest achievable spatial resolution in MRI, both being directly proportional to the signal-to-noise ratio (SNR) of the experiment.

The signal part of the SNR may be determined by the principle of reciprocity [3, 4], stating that there is a reciprocal relationship between the magnetic field at a defined point *x* in space, created by a unit current $\hat{I}$ running through a wire loop *w*_*l*_ on the one hand, and the induced electromotive force (emf *v*) along the wire loop *w*_*l*_, created by a rotating magnetic dipole (i.e., the sample) placed at the identical point *x* in space, on the other hand.

For a closed-current loop with a limited electrical resistance such as an NMR receiving coil, said induced electromotive force will result in heat dissipation that transforms into thermal noise, representing the main portion of the noise contribution of the SNR.

The SNR, first derived by Hoult and Richards [5], is given by [6]

$$\begin{array}{c} \text{SNR}=\frac{k_{0}\frac{B_{1}}{i}V_{\text{obs}}N\gamma \hbar^{2}I\left(I+1\right)\frac{\omega_{0}^{2}}{k_{\text{B}}T3\sqrt{2}}}{\sqrt{4k_{\text{B}}TR\Delta f}}\propto \frac{B_{1}}{i\sqrt{R}}=\frac{B_{1}}{\sqrt{P}}, \end{array}$$ (1)

where *k*_0_ is a scaling factor to account for the homogeneity of the radio-frequency (RF) coil employed, *B*_1_ is the RF-coil’s magnetic field strength, *i* the unit current, *B*_1_/*i* the coil’s sensitivity, *V*_obs_ the observed sample volume, *k*_B_ is the Boltzmann constant, *T* is the temperature of the coil and the sample, which is assumed to be identical, *N* the spin density, *I* the spin quantum number, ℏ is Planck’s constant *h* divided by 2*π*, *ω*_0_ = −*γB*_0_ is the Larmor frequency determined by the gyromagnetic ratio *γ* of the nucleus of interest and the strength of the static magnetic field *B*_0_, *R* is the electrical resistance of the coil contributing thermal noise, Δ*f* is the bandwidth of the receiver, and *P* the power.

The search for higher SNR has resulted in steadily increasing *B*_0_-field strengths, currently reaching a maximum of around 23.5 T (1 GHz ^1^H Larmor frequency) for commercially available NMR systems, while field strengths of human scale MRI magnets typically do not exceed 3 T in the clinic.

While both the noise level and the Boltzmann distribution would benefit from a reduced sample temperature, the freezing point and the operating temperature of the sample impose practical limits. Signal enhancement via non-equilibrium Boltzmann polarisation factors can also be reached through spin order transfer techniques such as dynamic nuclear polarisation (DNP) or parahydrogen induced polarisation (PHIP), which, however, often involve toxic substances and hence are not yet generally compatible with living biological samples.

Consequently, the SNR is maximised by using optimised hardware along the receive path, such as dedicated RF receiver coils. Such coil designs follow two strategies: (i) the filling factor [7] and hence the magnetic interaction is maximised when the coil geometrically conforms to the sample as closely as possible, and (ii) the sensitivity increases linearly as the diameter of the coil decreases for a constant height-to-diameter ratio [6]. The relative intrinsic SNR is therefore higher when using a smaller, sample-adapted coil. Hence such coils are used when recording MR images of body parts, e.g., the brain, or when acquiring spectra from rare or volume-limited substances [8]. However, situations remain where it is not possible to place the sample inside a dedicated active coil, such as when studying inner body parts. One remedy is to use a resonant inductively coupled miniaturised microcoil, as previously reported [9–11] and analysed in detail [12]. Of course, at the limit of miniaturisation of NMR resonators, this approach no longer works due to space limitations, especially for the more ideal case of adding capacitance towards achieving a resonant coupling structure [11].

To circumvent this issue, we introduce here a method to locally improve the sensitivity of the NMR (or MRI) experiment by means of broadband passive magnetic lenses. The working principle behind these lenses is geometry based and governed by Lenz’ law, and hence they are referred to as Lenz lenses [13].

**Fig 1:**
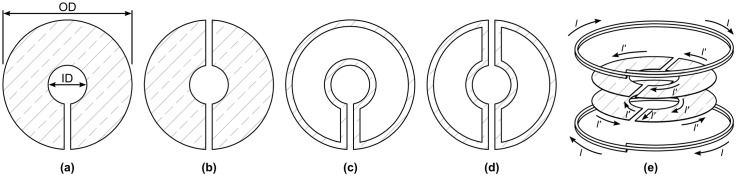
The four basic designs of Lenz lenses. They are either made from solid metal (subfigures (a) and (b)) or wire material (sub-figures (c) and (d)), arranged symmetrically ((b) and (d)) or non-symmetrically ((a) and (c)). The slit(s) guide induced current *I*′ from the outer edge to the inner edge, while reversing the flow direction, as further depicted in (e), where two lenses are arranged in parallel in a Helmholtz pair like configuration, denoted as a double Lenz lens configuration.

Lenz lenses are capable of focusing the magnetic field of a macroscopic RF coil into a smaller spatial region, thereby locally increasing the flux density for the same applied radiofrequency current. Lenz lenses can be simply made from wire, or conductive sheets such as copper foil, as shown in Fig 1, which illustrates two basic shapes.

Due to their simple geometry and wireless, broadband inductive coupling, Lenz lenses can be tailored easily to a particular application in a much more flexible manner than RF resonators. That is, Lenz lenses consist only of conductive loops, and are therefore straightforward to fabricate. Moreover, multiple lenses can be combined to enable the shaping or rotating of the magnetic field, similar to lenses in optics, and hence introduce a whole new degree of flexibility for the MR analysis of diverse samples. In particular, by reshaping the flux, our computations in the next section show that a Lenz lense can potentially achieve a higher degree of *B*_1_ uniformity than the outer excitation coil.

## Results

### Theory

Expressions are now derived for calculating the induced current within an arbitrary number of circular Lenz lenses made from wires and placed within the incident magnetic field generated by an external RF coil. Note that, by the theory of reciprocity, the expressions may be used in an equivalent sense to describe a situation in which a distinct volume of precessing magnetisation induces current within the Lenz lenses, which in turn induce current within the external RF coil. That is, the Lenz lenses and corresponding theory are applicable to both the excitation and receive chains of a conventional NMR (or MRI) experiment.

Consider the simple arrangement of placing a single circular Lenz lens coaxially at the midpoint between two elements of a Helmholtz pair. Faraday’s law of induction states that the electromotive force (emf), *ϵ*, generated in the lens is equal to the negative rate of change of magnetic flux Φ that it encloses:

$$\begin{array}{c} \epsilon =-\frac{\partial \Phi }{\partial t}. \end{array}$$ (2)

Lenz’s law states that the sense of the current induced is such that it opposes this flux, hence the minus sign in Eq (2). In the limit that the gap size between the elements connecting the outer and inner loops of the lens is zero, the flux impressed on the lens is equal to that enclosed by the outer loop minus that enclosed by the inner loop:

$$\begin{array}{c} \Phi =2\left(M_{BH}-M_{SH}\right)I_{H}, \end{array}$$ (3)

where *I*_*H*_ is the current in the Helmholtz pair, *M*_*BH*_ is the mutual inductance between the outer loop of the lens (*B* ≡ big) and one element of the Helmholtz pair, and a similar definition holds for *M*_*SH*_ (*S* ≡ small). We ignore capacitive effects and model the Lenz lens itself as an *RL*-circuit:

$$\begin{array}{c} V=I_{L}R_{L}+L_{L}\frac{dI_{L}}{dt}, \end{array}$$ (4)

where *L*_*L*_ = *L*_*S*_ + *L*_*B*_ − 2*M*_*SB*_ is the self-inductance of the lens (with corresponding definitions for *L*_*S*_, *L*_*B*_ and *M*_*SB*_), *R*_*L*_ is the resistance of the lens and *I*_*L*_ is the induced current. Equating the potential in Eq (4) to the emf in Eq (2) (combined with Eq (3)) and assuming a time-harmonic regime, we obtain the following expression for the induced current within the lens:

$$\begin{array}{c} I_{L}=\frac{2i\omega \left(M_{BH}-M_{SH}\right)I_{H}}{\left[R_{L}-i\omega \left(L_{S}+L_{B}-2M_{SB}\right)\right]}, \end{array}$$ (5)

where *ω* is the operating frequency (radians/s) of the RF coil. Eq (5) represents a generalisation of an expression provided by Schoenmaker et al. [13], which was derived for a Lenz lens operating in the kilohertz regime. Those authors ignored the resistance term in Eq (5), hence neglecting the frequency dependence of the induced current, and they also ignored the mutual inductance between the inner and outer loops of the lens.

For high-frequency applications, such as the megahertz regime relevant to NMR, it is necessary to consider the skin-effect in the resistance and inductance calculations used in Eq (5). Let us label the resistance of a wire under direct current to be *R*_0_ and under (high-frequency) alternating current to be *R*_*L*_, as above. The ratio *R*_*L*_/*R*_0_ can be calculated exactly using Kelvin-Bessel functions [14], however this approach is computationally unstable when the skin-depth is small relative to the wire radius. A common alternative is to approximate the relative increase in resistance by the inverse of the relative decrease in the effective cross-sectional area of the ring defined by the skin-depth [15]. This approach is accurate to within 5.5% of the exact result when the ratio of wire radius, *a*, to skin-depth, *δ*, is greater than one [16], and below this limit the resistance is simply equal to *R*_0_:

$$\begin{array}{c} \frac{R_{L}}{R_{0}}=\left\{ \begin{array}{cc} 1, & 0\leq \frac{a}{\delta }<1\\ \frac{a^{2}}{2a\delta -\delta^{2}}, & \frac{a}{\delta }\geq 1, \end{array}\right. \end{array}$$ (6)

where *R*_0_ = *ρ*[2*πr*_*B*_ + 2*πr*_*S*_ + 2(*r*_*B*_ − *r*_*S*_)]/(*πa*^2^), in which *r*_*B*_ is the radius of the outer loop of the lens and *r*_*S*_ is the radius of the inner loop, $\delta =\sqrt{2\rho /\omega \mu }$, and *ρ* and *μ* are the resistivity and permeability of the conductor, respectively (e.g. for copper 1.68 × 10^-8^ Ωm and *μ* ≈ *μ*_0_ = 4*π* × 10^−7^ Hm^−1^).

The self-inductance terms, *L*_*B*_ and *L*_*S*_, may be calculated using the approximation [17]:

$$\begin{array}{c} L\approx \mu_{0}r\left[\ln\left(\frac{8r}{a}\right)-2+\frac{Y}{2}\right], \end{array}$$ (7)

where *r* is the radius of the loop in question and the parameter *Y* depends on the frequency of operation. *Y* = 0 is the high-frequency limit for which current is restricted to the surface of the wire and *Y* = 1/2 is the low-frequency limit for which the current is uniform throughout the wire; hence we set *Y* = *R*_0_/(2*R*_*L*_) (see Eq (6)). Note that Eq (7) is accurate to *O*(*a*^2^/*r*^2^) [17].

The mutual inductances between the Helmholtz coil and the elements of the Lenz lens, and between the lens elements themselves, can be calculated using the formulae provided by Babic et al. [18] (i.e. Eqs (24)-(25)). These formulae were derived following a vector potential argument and are applicable to any pair of arbitrarily placed circular conductors. Note that Matlab code for evaluating these formulae has been made available by Babic et al. on their publisher’s website (http://ieeexplore.ieee.org).

Let us now consider the general case of multiple lenses placed arbitrarily within the vicinity of a Helmholtz pair, as depicted in Fig 1e. We must now treat the two loops of the Helmholtz pair separately (subscripts *H*1 and *H*2) and consider also the mutual inductances that exist between the elements of different lenses. After careful consideration of the enforced current sense between inner and outer loops of each lens, we arrive at the following expression for the flux contained by the *k*th lens (*k* = 1:*K*):

$$\begin{array}{ccc} \Phi_{k} & = & \left(M_{BkH1}+M_{BkH2}-M_{SkH1}-M_{SkH2}\right)I_{H}\\ & & +\sum_{j=1,j\neq k}^{K}\left(M_{BkBj}-M_{SkBj}-M_{BkSj}+M_{SkSj}\right)I_{Lj}, \end{array}$$ (8)

where, for example, *M*_*BkSj*_ is the mutual inductance between the outer loop of the *k*th lens and the inner loop of the *j*th lens, and *I*_*Lj*_ is the induced current in the *j*th lens. Similarly, the potential for the *k*th lens is given by:

$$\begin{array}{c} V_{k}=\left[R_{Lk}-i\omega \left(L_{Sk}+L_{Bk}-2M_{SkBk}\right)\right]I_{Lk}. \end{array}$$ (9)

Combining Eqs (2), (8) and (9) and rearranging, we obtain the following matrix equation:

$$\begin{array}{c} LI_{L}=H, \end{array}$$ (10)

where

$$\begin{array}{c} \begin{array}{ccc} L_{kk} & = & R_{Lk}-i\omega \left(L_{Sk}+L_{Bk}-2M_{SkBk}\right)\\ L_{kj} & = & -i\omega (M_{BkBj}-M_{SkBj}-M_{BkSj}+M_{SkSj})\\ H_{k} & = & i\omega \left(M_{BkH1}+M_{BkH2}-M_{SkH1}-M_{SkH2}\right)I_{H} \end{array}\;\left(\begin{array}{c} k=1:K\\ j=1:K\\ j\neq k \end{array}\right), \end{array}$$ (11)

and $I_{L}$ is a vector of length *K*, which contains the induced current for each lens. Note that it is straightforward to show that Eqs (10) and (11) reduce to Eq (5) for the case of a single symmetrically placed lens.

### Simulations and optimisation

Eqs (10) and (11) permit the investigation of the dependence of the induced current(s) and corresponding magnetic field on a variety of design parameters, such as the number of lenses, their geometry and the frequency of operation. As an illustrative example, Eq (10) was solved for several different arrangements appropriate to the experiments described in *’MRI experiments’*. That is, we considered a Helmholtz pair with a radius of 0.6 mm and a separation of 0.6 mm, carrying a current of 66 mA at 500 MHz. The wire for the lens(es) was modelled using an effective circular cross-section with radius *a* = 8.9 μm. The radius of the outer loop of the lens(es) was fixed at *r*_*B*_ = 0.5 mm for all cases. The radius of the inner loop of the lens(es), *r*_*S*_, was set at either 0.1 mm or 0.2 mm and we considered both single lens and double lens cases, with a lens separation for the latter of either 0.1 mm or 0.2 mm. All simulations were performed using Matlab^®^ (R2012b, Mathworks^®^).

In general, a smaller radius for the inner loop of a lens results in a stronger magnetic field at the midpoint. However, in NMR (or MRI) it is desirable not only to have a strong transmit RF field (and correspondingly high receive sensitivity) but also to have a homogeneous field, such that the flip angle is constant throughout the region of interest (ROI). Therefore the design of the Lenz lens(es) for NMR becomes an optimisation problem with the goal of maximising the induced field within the ROI, with respect to the lens geometry, while maintaining an acceptable level of homogeneity. That is, we wish to solve:

$$\begin{array}{cc} & \max_{\rho }\left\{{\text{mean}}_{\text{ROI}}\left\{B_{z}\left(\rho \right)\right\}\right\}\;\text{s.t.}\\ & \;{\text{mean}}_{\text{ROI}}\{∥B_{z}\left(\rho \right)-{\text{mean}}_{\text{ROI}}\left\{B_{z}\left(\rho \right)\right\}∥\}\leq \epsilon , \end{array}$$ (12)

where ***ρ*** is a vector containing the radii of the inner and outer loops and the axial positions of each lens, *B*_*z*_ is the axial component of the total magnetic field calculated using the Biot Savart law, and *ϵ* is the specified acceptable average field error. As an illustrative example in keeping with the simulations outlined above, the ROI was chosen to be a cylindrical volume of radius 0.06 mm and height 0.06 mm, centred at the origin and coaxial with the lenses and Helmholtz coil. The mean terms in Eq (12) were evaluated by integrating numerically (trapezoidal rule) over the ROI and then dividing by the volume.

The optimisation in Eq (12) was achieved using the function fmincon from the Matlab^®^ Optimisation Toolbox™ (interior-point algorithm) for three cases: one, two and four lenses. To improve convergence for the cases with multiple lenses, the optimisation was performed in two stages: firstly, the axial placements of the lenses were fixed at equal increments between the Helmholtz coils and Eq (12) was solved with respect to the lens radii alone; secondly, the results from the first optimisation were used as the starting guess for the full optimisation of Eq (12). Additional constraints were imposed to ensure that: the inner loops were always smaller than the outer loops; the axial placements of multiple lenses were always separated by at least the wire diameter; the axial placements of pairs of multiple lenses were always symmetric about *z* = 0 and closer to the origin than the Helmholtz coils; the radii of the outer loops were always less than twice that of the Helmholtz coil (and all radii were positive). The optimisations took the order of seconds (1 lens, 7 s; 2 lenses, 25 s; 4 lenses, 106 s) on a standard desktop computer (2.7 GHz Intel^®^ Core™ i7 processor with 16 GB of RAM).

**Fig 2:**
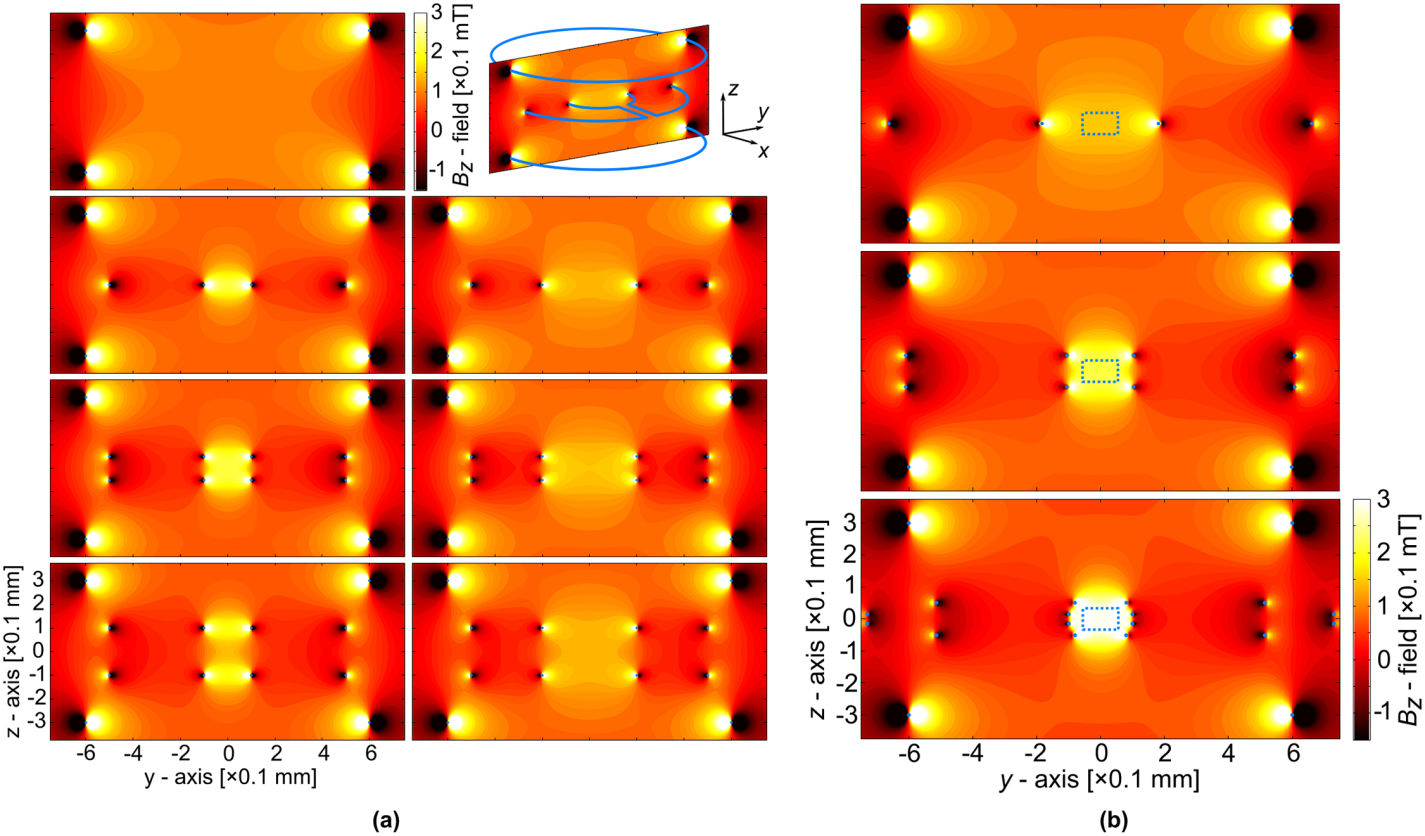
Simulated distribution of the *z*-component, *B*_*z*_, of the *B*_1_ magnetic flux density for different geometrical arrangements and optimisation of the magnetic field’s homogeneity. Note that the *B*_0_-field is oriented parallel to the planes of the coil pair and the lenses (e.g. along the *x* or *y*-axis). (a) The *B*_*z*_-field induced by a Helmholtz pair and a variety of different lenses: i.e. none, one or two lenses of different inner loop size and/or axial position. The colour contour plots are all plotted on the *yz*-plane as depicted in the illustrative example at the upper right of the figure. (b) Three optimised systems consisting of a Helmholtz pair and either one, two or four lenses. The size and position of the lens elements have been optimised to maximise the amplification within a cylindrical volume, depicted in the *yz*-plane by the blue dashed rectangle, whilst constraining the average field error to be 1% within this region of interest. The example with four lenses exhibits a threefold amplification of field sensitivity.

Fig 2a displays colour contour plots of the simulated *z*-component of the induced magnetic field over the plane *x* = 0 for several different arrangements of Lenz lenses within the Helmholtz pair. Similarly, Fig 2b displays the results of solving the optimisation problem defined by Eq (12) for the cases of one, two and four lenses.

### MRI experiments

Symmetrical wire and plate-based Lenz lenses according to Fig 1b and 1d with 1.0 mm outer diameter (OD), and inner diameters (IDs) of 0.2 mm and 0.4 mm were patterned by means of photo-lithography and electroplating on glass substrates to be used with a custom micro Helmholtz coil pair. The four designs fabricated are summarised in Table 1, and short identifiers were assigned for easier discrimination of the different designs. Note that a Helmholtz pair was chosen for convenience in the present work, with regards to fabrication simplicity, lens positioning and observation. Furthermore, the extensive uniform field induced by a Helmholtz pair permitted straightforward analysis of any field variation caused by the lenses.

**Table 1:** Overview of the four different double Lenz lens variants manufactured.

Type	Reference	OD (mm)	ID (mm)	Identifier
Plate	Fig 1b	1.0	0.2	LL1
			0.4	LL2
Wire	Fig 1d	1.0	0.2	LL3
			0.4	LL4

To quantify the amplification of the lenses and to verify the theory developed above, we acquired a series of spin echo imaging experiments using a similar 1.2 mm diameter micro Helmholtz coil pair setup as presented in [19], but with one instead of two coil windings on each side. The Lenz lens designs LL1-LL4 were primed with deionized water and arranged in the setup illustrated in Fig 1e before MRI was performed at steadily decreasing attenuations. For reference, we further acquired MR images without a Lenz lens present using comparable chips filled with H_2_O. A photograph of the actual setup and a brief summary of the results obtained is presented in S1 Fig.

Pulse parameters for the individual designs were determined by exporting the absolute signal values from ParaVision using a macro (Bruker). Keeping the pulse length *τ* constant, the *B*_1_-field required to generate a flip angle *α* for a specific nucleus is given by

$$\begin{array}{c} B_{1}=\frac{\alpha }{\gamma \tau }, \end{array}$$ (13)

which leads to *B*_1_ = 0.53mT for *τ* = 11 *μ*s, and *α* = *π*/2 (90°) using the proton gyromagnetic ratio *γ*(^1^ *H*) = 267.513 × 10^6^ rad s^−1^T^−1^.

However, due to the non-uniform field profile of the lens, a mixture of flip angles is generated. A global 90°-pulse was defined by locating the highest signal amplitude along the sweeped power range, although the local flip angle might be ≤90° at these particular pulse parameters.

The probe efficiency *η*_p_, i.e., the conversion efficiency of power into magnetic field for a certain probe (Lenz lens) is given by

$$\begin{array}{c} \eta_{\text{p}}=\frac{B_{1}}{\sqrt{P}} \end{array}$$ (14)

and therefore *η*_p_ ∝ SNR according to Eq (1).

**Fig 3:**
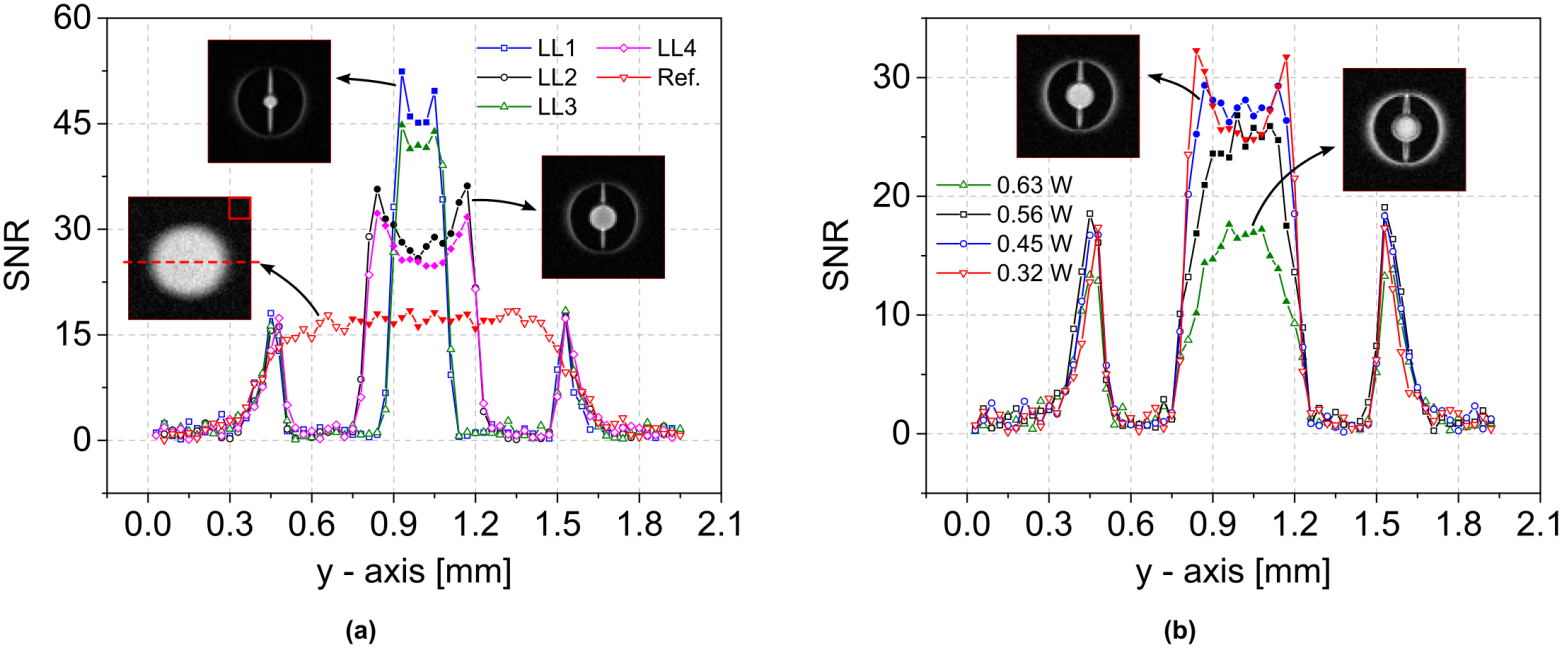
Obtained SNR line profiles from the different designs and at different excitation powers. (a) Extracted SNR line profiles of LL1-LL4 and of the reference scan along the *y*-axis, as exemplified by the broken line in the reference image. All scans were acquired at a global flip angle of 90°. The mean noise was calculated from a 12 × 12 pixel matrix in the upper right corner of the same scan, as depicted. Statistics were derived from the regions illustrated by filled symbols. The powers for LL1 to LL4 and the reference scan were 0.18 W, 0.25 W, 0.18 W, 0.32 W and 1 W. (b) SNR profiles of LL4 at global flip angles of 90°, 106°, 119° and 126° and corresponding pulse powers of 0.32 W, 0.45 W, 0.56 W and 0.63 W. Statistics were derived from the regions illustrated by filled symbols.

Fig 3a presents SNR line profiles along the horizontal *y*-axis for LL1-LL4 and the reference scan without a lens. The SNR was calculated by taking the ratios of the exported absolute values and the mean noise value obtained from a 12 × 12 pixel matrix from the reference scan, as indicated in the figure. The line profiles were extracted from the images that were acquired using the individual, global 90°-pulse parameters evaluated for each type. For LL1-LL4 the determined powers *P*_*i*_ were 0.18 W, 0.25 W, 0.18 and 0.32 W, while for the reference scan, the 90°-pulse was found at *P*_0_ = 1 W. These values lead to probe efficiencies *η*_p,*i*_ of 1.25 MT W^−1/2^, 1.06 MT W^−1/2^, 1.25 MT W^−1/2^ and 0.94 MT W^−1/2^, for LL1-LL4 respectively, while the reference efficiency was *η*_*p*,0_ = 0.53mT W^−1/2^.

Note that the non-uniform field profile associated with each lens leads to a local sinusoidal modulation of the flip angle while sweeping the power. As such, while the global 90°-pulse defined above results in the highest achievable signal amplitude, it does not lead to the highest achievable signal uniformity within the ROI. The evolution of the SNR profile of LL4 for *α* = 90°, 106°, 119° and 126° is illustrated in Fig 3b, where *α* = 90° was found at 0.32 W, and the profile clearly changes from convex to concave shape as the power is increased.

The experimental results shown in Fig 3a and 3b are summarised in Table 2, which furthermore contains calculated figures of merit. Data points defining the regions of interest, which were taken into account for calculations, are marked with filled symbols in both figures, while those values outside the ROIs are represented by hollow symbols.

**Table 2:** Calculated mean, maximum, and standard deviation (STD) of the SNR values from Fig 3a and 3b as well as derived figures of merit.

Design	*i* *	*P*_*i*_ (W)	*α* ^†^	Mean	Max	STD	$n_{i}^{‡}$	$\sqrt{P_{0}/P_{i}}$	$M_{i}^{§}$	*η*_p,*i*_ $\left(\frac{\text{mT}}{\sqrt{\text{W}}}\right)$	*η*_eff,*i*_ $\left(\frac{\text{mT}}{\sqrt{\text{W}}}\right)$ ^¶^	*V*_*i*_/*V*_0_ ^#^
Ref.	0	1.00	90°	17.2	18.4	0.7	18	1.0	1.0	0.53	0.53	1
LL1	1	0.18	90°	47.7	52.4	3.2	5	2.4	2.8	1.25	1.48	1/36
LL2	2	0.25	90°	30.2	36.2	3.4	12	2.0	1.8	1.06	0.95	1/9
LL3	3	0.18	90°	42.7	44.8	1.5	5	2.4	2.5	1.25	1.33	1/36
LL4	4	0.32	90°	27.5	32.3	2.8	12	1.8	1.6	0.94	0.85	1/9
LL4	5	0.45	106°	24.5	29.3	1.2	12	1.5	1.4	0.94	0.74	1/9
LL4	6	0.56	119°	23.2	26.8	3.2	12	1.3	1.3	0.94	0.69	1/9
LL4	7	0.63	126°	15.0	17.6	2.4	12	1.3	0.9	0.94	0.48	1/9

* Running index number.

^†^ A global *α* of 90° was assumed at the maximum peak SNR amplitude. Deviating flip angles were calculated based on *η*_p,*i*_ and by combining Eqs (13) and (14).

^‡^ Number of voxels *n*_*i*_ taken to calculate mean, max and STD. The regions taken into account are represented by filled symbols in Fig 3a and 3b.

^§^ Enhancement *M*_*i*_ based on the ratio of the mean SNR values with respect to the reference scan without any lens.

^¶^ Effective probe efficiency *η*_eff,*i*_ = *M*_*i*_ ⋅ *η*_p,0_ with respect to the reference scan probe efficiency and the measured enhancements *M*_*i*_.

^#^ Ratio of the observable volumes of interest *V*_*i*_ based on a constant slice thickness and on the various IDs with respect to the reference scan without any lens.

### Comparison between model and experiment

**Fig 4:**
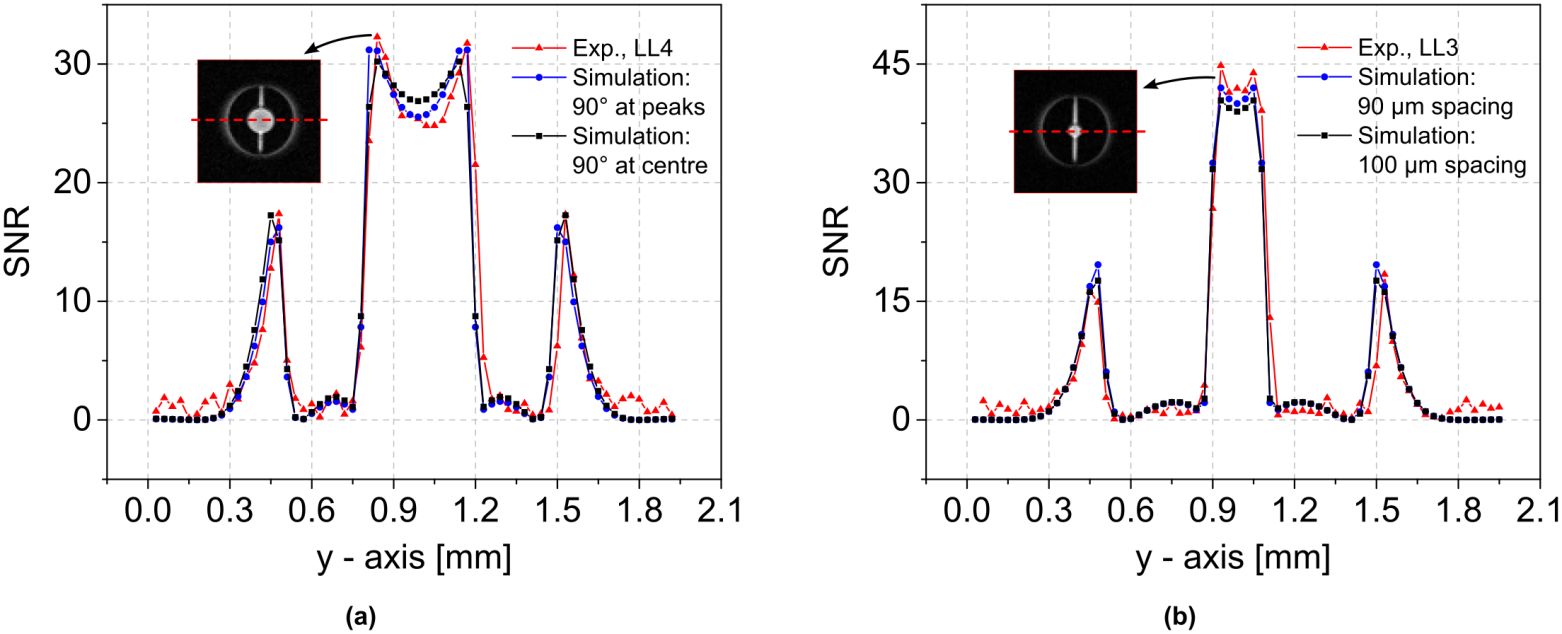
SNR line profiles to compare the measurements and the model developed above as well as to verify the influence of design parameters. (a) Comparison of the measured SNR profile for LL4 and the simulated SNR profiles for two differently defined 90°-pulses: at the highest signal intensity in accordance with the experiment; at the centre-point of the arrangement. (b) Measured and simulated SNR profiles for LL3 at the 90°-pulse power. Simulated profiles were calculated for two different lens spacings (90 μm and 100 μm), while in both cases, the 90°-pulse was defined at the centre-point.

Fig 4a and 4b present comparisons between the experimental results and the corresponding simulations according to Eqs (10) and (11). The simulated SNR profiles were generated by first calculating the field amplification *B*_*zA*_ over an array of 31 × 65 × 101 points: 31 points across the pixel in the *x*-direction, 65 points along the *y*-axis (i.e., one for each pixel), and 101 points across the slice in the *z*-direction. This field was averaged over the *x*- and *z*-directions, and the value (*B*_*zA*90_) either at the highest signal peak or at the centre-point in *y* was used to define the 90° flip angle. A corresponding SNR array of 31 × 65 × 101 points was then generated by applying SNR = |(*B*_*zA*_ sin(*π*/2 ⋅ *B*_*zA*_/*B*_*zA*90_))|. This SNR array was subsequently averaged over the *x*- and *z*-directions to give the SNR amplification factor along the *y*-axis. Finally, the result was multiplied by the maximum SNR of the reference scan, listed in the first row of Table 2.

Note that this approach ignores any possible additional losses incurred by the lens itself. Accounting for the skin-effect at 500 MHz, the resistances of the wire-based double lenses used in the experiment are approximately equal to that of the Helmholtz pair. However, since the induced currents differ considerably, the power dissipation in the lenses is approximately 15-20 times smaller than that in the Helmholtz coil, and hence these losses were assumed to be negligible. The fact that we achieve such a good match between theory and experiment, and that the results for the wire-based and plate-based (lower resistance) lenses are so similar, confirms the validity of this assumption. A detailed analysis of the noise characteristics of Lenz lenses is important, but such an analysis is beyond the scope of the present proof-of-concepts study and has been left for future work (cf. Jacquinot and Sakellariou [12]).

Fig 4a displays SNR profiles for LL4 and demonstrates the impact of defining the global 90°-pulse either at the location of the largest field amplification (as per the experiment) or at the centre-point (cf. Fig 3b). Fig 4b displays SNR profiles for LL3 and demonstrates the impact of error in the spacing of the lenses (i.e. 100 μm versus 90 μm). For both of the simulated profiles in Fig 4b, the 90°-pulse was defined at the centre-point, and for the case with 90 μm spacing the array size used in the calculation was reduced to 31 × 65 × 91 points.

## Discussion

Fig 2a demonstrates that the magnitude and homogeneity of the focused field is strongly dependent on the number and geometrical parameters of the Lenz lenses. For a single lens, a smaller inner loop results in a stronger field at the cost of homogeneity. However, with the introduction of a second lens this homogeneity can be partially recovered, provided that an appropriate axial separation is chosen. For example, those pairs of lenses in Fig 2a for which the ratio of the inner loop radius to the axial separation is equal to one (i.e. akin to the Helmholtz pair; column 1, row 3 and column 2, row 4) clearly display a superior trade-off of amplification to homogeneity, over a spherical ROI, compared to the other arrangements.

However, to obtain the best trade-off over an arbitrarily shaped ROI, especially when considering additional lenses, optimisation via Eq (12) is necessary. Fig 2b demonstrates that for an average relative field error of 1% within the ROI defined in *‘Simulations and optimisation’*, it is possible to achieve amplifications of 1.5, 2.2 and 3.0 with one, two and four lenses, respectively, over the use of a Helmholtz pair alone. If the field constraint is relaxed to 2% error, the amplification increases to 1.7, 2.5 and 3.2, respectively. Notice that for the case of four lenses in Fig 2b, the inner loops lie approximately on the surface of a sphere. However, for the equivalent case with 2% error, the two pairs of lenses are brought into close proximity to one another.

Eqs (10)–(12) therefore allow the study and design of a wide variety of lens arrangements with flexible field constraints. Indeed they may be applied to circular lenses of any orientation without necessarily being restricted to the centred coaxial arrangements considered herein. Furthermore, the choice of considering a Helmholtz pair as the transmit/receive RF coil has been made for demonstrative purposes only, since it was also used in some of the experiments, and the theory is applicable to any system for which the mutual inductance can be calculated. Note that we have ignored capacitive effects that may be present for multiple closely-spaced lenses and also the finite gap between the elements that join the inner and outer loops. Both of these factors will reduce the achievable amplification by some degree. Note also that a direct method was used to solve Eq (12), which was somewhat sensitive to the initial guess and hence the two-stage procedure described in *’Simulations and optimisation’* was implemented. An alternative approach would be to use stochastic optimisation, such as simulated annealing, to guarantee convergence to the global optimum at the cost of runtime.

The experiments performed in this study clearly confirm the focusing effect and the associated local SNR enhancement as predicted by the model. For the designs fabricated in this study, we achieved enhancements 1.6 ≤ *M*_*i*_ ≤ 2.8 (Table 2). Higher SNRs were achieved for the plate-based designs due to the lower electrical resistance, i.e., the increased noise contribution in the case of the wire-based design. For the largest enhancement *M*_1_, the power required to generate a 90°-pulse was reduced by a factor of 5.6 compared to the reference. Although in this case the observable volume of interest was reduced to 1/36 of the initial volume, the gain in SNR corresponds to a 2.8^2^ ≈ 7.8-fold decrease in acquisition time, since $\text{SNR}\propto \sqrt{\text{TA}}$ [20]. In other words, the use of an LL1 enables samples with 1/8 concentration to be inspected, without altering the SNR or acquisition time, in comparison to the situation without a lens present. This circumstance may be especially helpful for scenarios in which sample dimensions and concentrations are fixed, e.g., when studying small organisms such as insects.

Mean SNR enhancement may be estimated using the derived probe efficiencies *η*_p,*i*_, which are based on the 90°-pulse power *P*_*i*_, and these were found to correspond well with the measured effective probe efficiencies *η*_eff,*i*_ (Table 2). Note that if it were possible to build an active Helmholtz pair with diameter 0.2 mm, one would expect a probe efficiency between 1.17 mT w^−1/2^ and 2.65 mT w^−1/2^, after applying a scaling law to the present design (diameter 1 mm) based on the inverse or inverse square root of the coil diameter [6]. In comparison, the measured probe efficiencies for LL1 and LL3 were 1.48 mT w^−1/2^ and 1.33 mT w^−1/2^, respectively, which further demonstrates that Lenz lenses represent a viable alternative to reducing coil size, particularly in cases where such a reduction is either difficult or impossible.

A trade-off between the enhancement and the homogeneity of the SNR profile was clearly apparent in the experimental results. For example, for LL4 at *α*_4_ = 90° we observed an enhancement of *M*_4_ = 1.6 and a standard deviation (STD) of 2.8, whereas at *α*_5_ = 106° the STD improved by 57% to 1.2 at the cost of a 14% decrease in the enhancement to *M*_5_ = 1.4 (see also Fig 3b). In the latter case, *α* approached 90° at the centre-point and exceeded 90° at the peak field positions, which occurred close to the inner edge of the Lenz lens defined by the inner diameter, and this resulted in a reduced SNR at these points.

The observed behaviour in the experiments is matched well by the theoretical model, as illustrated in Fig 4a for LL4. For the case in which the 90°-pulse corresponds to the peak field positions, the simulations are in high agreement with the results obtained in the experiment. Furthermore, the simulated profile flattens out for the case in which the 90°-pulse is at the centre-point, as expected from the experiments, and STD decreases from 2.3 to 1.3.

Good agreement between simulations and experiment was also found for LL3, as shown in Fig 4b. The slightly higher SNR values obtained in the experiment may be a result of fabrication tolerances, such as uncertainty in the spacing between lenses. In the present case, such tolerances may occur if the final thickness of the plated lenses is either not uniform or inaccurate, or if the thickness of the photoresist defining the spacing between the two lenses deviates from its nominal value. The example given in Fig 4b shows that reducing the spacing from 100 μm and 90 μm leads to a 3% increase in mean SNR. Furthermore, the simulations assume line currents for the field calculation, whereas LL3 and LL4 are constructed using conductive material with a rectangular cross-section that has a low aspect ratio. Therefore, the simulations may represent a lower bound to the trend observed in Fig 3a from plate-based to wire-based lenses.

## Conclusion

We have presented a novel method to locally increase the signal-to-noise ratio in nuclear magnetic resonance by using Lenz lenses to focus the RF magnetic field of an NMR coil into a smaller volume of interest. As a result, higher spatial resolutions or reduced acquisition times become possible. The achievable enhancement strongly depends on the size ratios of the excitation coil and the dimensioning of the lens. The use of Lenz lenses enabled not only the magnetic flux density to be intensified, but also the resulting field to be shaped by arranging multiple lenses in a pre-defined manner. The paper presented a theoretical description of a set of Lenz lenses, which conformed closely to the experimental data obtained in a series of imaging experiments. The simulations enabled the optimisation of the design with respect to parameters such as signal amplitude and homogeneity. Lenz lenses are useful not only for amplifying the *B*_1_ magnetic field in the sample, but also for attenuating it in other regions, for example to avoid exciting regions with jumps in magnetic susceptibility, which would otherwise lead to distortions of the experiment, or to protect electronics from strong RF radiation.

The Lenz lenses are broadband, due to their low intrinsic capacitance and inductance, and hence do not detune the outer coil. In the present work, this effect was not exploited directly (for example in heteronuclear experiments) since we focused on proton MRI. Nevertheless, the broadband nature of the lenses ensures that they do not generate any additional susceptibility induced frequency shifts that may otherwise be caused by material inhomogeneities or capacitive loading.

Flux guiding has in the past also been implemented by so-called magneto-inductive guides in the form of RF metamaterials, for example to span the gap between an MRI sensor and the region of interest to be imaged [21–24]. In this sense, there is a similarity in purpose of the metamaterial unit cell and a Lenz lens. The key difference between the two approaches is the suppression of resonance in the case of Lenz lenses, i.e., the introduction of a significant amount capacitance is completely avoided in a Lenz lens structure. As an outlook, Lenz lenses may be a possible choice for magneto-inductive guides to overcome larger distances, as non-resonant unit cell metamaterial structures, an aspect that was not considered in the present work.

Lenz lenses have a further useful characteristic that greatly benefits both NMR detection and miniaturisation. Since a Lenz lens gathers magnetic flux from its closed loop, exactly cancelling the flux in this region, and deposits this flux within its anti-winding, there is no stray NMR excitation from materials directly outside of the anti-winding. For NMR analysis, this means that the detected signal only emanates from the sample and not from the sample container materials, and is therefore uncluttered by spectral peaks from the chip materials. For miniaturisation, this means that a larger class of “building” materials become available to construct NMR-compatible micro-devices, for example soft polymers, especially those that would otherwise disturb the detected NMR spectra by contributing strong and unwanted spectral responses.

## Materials and methods

### Simulations

All simulations were performed using Matlab^®^ (R2012b, Mathworks^®^). The optimisation in Eq (12) was achieved using the function *fmincon* from the Matlab^®^ Optimisation Toolbox™ (interior-point algorithm) for three cases: one, two and four lenses. The optimisations took the order of seconds (1 lens, 7 s; 2 lenses, 25 s; 4 lenses, 106 s) on a standard desktop computer (2.7 GHz Intel^®^ Core™i7 processor with 16 GB of RAM).

### Device fabrication

**Fig 5:**
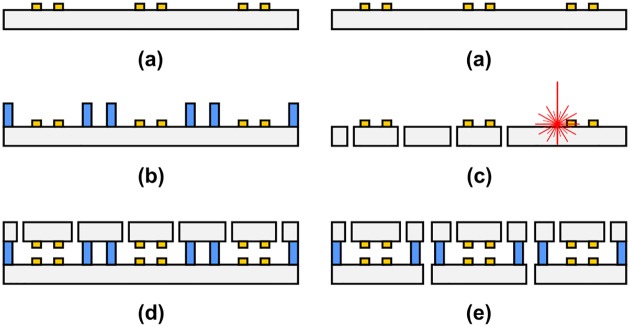
Fabrication steps of the double Lenz lens chips. (a) Au-electroplating of lenses on two glass substrates. (b) Lamination and structuring of dry film photoresist on first wafer. (c) UV-laser drilling of microfluidic ports into second wafer. (d) Adhesive full-wafer bonding of both substrates. (e) Dicing of individual chips.

Fig 5 illustrates the fabrication steps of the double Lenz lens chips used for the experiments.

At first, a 15/150 nm Cr/Au seed layer was evaporated on two 208±20μm thick, 4-inch diameter float glass wafers (D 263^®^ T, Schott AG, Mainz, Germany), where the Cr-layer served as an adhesion promoter between the glass surface and the Au-layer. Subsequently, Hexamethyldisilazane (HMDS) was applied from the gas phase before spin-coating a 20 μm layer of AZ9260 positive-tone photoresist (MicroChemicals GmbH, Germany). The wafers were stored in ambient atmosphere for around 4 h for rehydration, before windows for electroplating were opened using UV photo-lithography, which served as a mould structure. In the subsequent electroplating step, alignment marks and Lenz lenses were structured accordingly by depositing a 5 μm thick Au-layer. In the case of the wire-type lenses, the width of the conductor was 50 μm. An SEM-closeup of an electroplated, wire-type Lenz lens is presented in S2 Fig.

Afterwards, the mould layers were stripped using acetone before the Au seed layer was etched using a potassium iodine/iodide based etchant. All etching and stripping steps were performed using megasonic agitation to ensure uniform wetting of the surface and homogeneous etching rates.

Before the subsequent UV-laser drilling step, dicing foil was laminated on both sides of one wafer to protect the substrate from debris during laser ablation. The latter was done using a UV-laser (TruMark 6330, Trumpf, Germany), where 108 holes were drilled to realise two microfluidic ports for each of the 54 chips. After drilling, the fragile wafer was placed in a petri dish with isopropyl alcohol (IPA), which dissolved the adhesive of the dicing foil, before the wafer was rinsed in deionized (DI) water and dried using nitrogen gas.

Two layers of Ordyl SY355 dry film resist (Elga Europe s.r.l., Milano, Italy) were laminated onto the second substrate using a hot roll laminator (Mylam 12, GMP, Polch, Germany). Lamination was done at a speed of 1 cm s^−1^, a pressure of 1 bar, and a temperature of approximately 100°C, resulting in a total nominal resist-thickness of 110 μm. The resist was UV exposed at 180mJ cm^−2^ using a mask aligner (MA6, Karl Suss, Germany) to pattern microfluidic channels. After exposure, a post-exposure bake (PEB) was performed for 1 min at 85°C. The structures were developed for about 6 min in BMR developer using ultrasonic agitation, followed by rinsing in IPA and DI water. Finally, the wafer was spin-dried.

Both substrates were subsequently bonded in a full wafer bonding process [25, 26] using a substrate bonder (SB6, Karl Süss, Germany) to realise the double Lenz lens configuration, i.e., parallel pairs of lenses. The wafers were aligned manually and fixed using the clamps of the SB6 chuck before loading it into the machine, where a tool pressure of 2.4 bar was applied for 30 min at 95°C. The stack was hard baked for 2 h at 150°C before dicing 54 chips with a nominal size of 5 × 16 × 0.5 mm (width × length × height). The resulting spacing between the lenses of ≈100μm was hence defined by the thickness of the deposited Au-layer and the height of the previously patterned Ordyl photoresist layers.

### Magnetic resonance imaging

All magnetic resonance imaging experiments were performed on an Avance III NMR system (Bruker, Rheinstetten, Germany), controlled by the ParaVision^®^ 5.1 imaging software (Bruker). The NMR scanner was operated at the proton Larmor frequency of 500.13 MHz in combination with a Micro 5 micro-imaging probe base and a Micro 5 gradient system, driven at 40 A, which results in a maximum gradient strength of 2 Tm^−1^.

Throughout the experiments, the attenuation (ATT) was varied from 70 dB to 50 dB (0.025W ≤ *P* ≤ 2.5W) in steps of 0.5 dB. The relationship between ATT and *P* is given by

$$\begin{array}{c} P=10^{\frac{{\text{ATT}}_{0}-\text{ATT}}{10\;\text{dB}}}P_{0}, \end{array}$$ (15)

where *P*_0_ = 1 W and ATT_0_ = 54 dB. Acquisition parameters were set to: repetition time TR = 500 ms, echo time TE = 5.3 ms, flip angle *α* = 90°, effective slice thickness SI = 100 μm, field of view FOV = (1.92mm)^2^, matrix MTX = 64 × 64 and hence 30 × 30μm^−2^ in-plane resolution, number of averages NEX = 4 and an acquisition time (TA) per scan of 2 min 8 s.

Obtained MR reference images without any Lenz lens present are given in S3 Fig, while the acquired image sequences for LL1-LL4 are depicted in S4–S7 Figs. The effect of varying pulse power is clearly visible in these four images, from which the optimal power can be derived by inspection.

## Supporting information

S1 Fig Overview of the experimental setup and summary of the most significant MR images acquired. (a) Photograph of a LL3 chip inserted in between the 1.2 mm diameter micro Helmholtz coil pair used throughout the experiments. The microfluidic chamber filled with DI-water is depicted by broken red lines. (b) Acquired MR image using a LL4 chip with a 90° pulse at 0.32 W. (c) 90° reference scan without Lenz lens chip, which required a threefold higher power of 1.00 W. (d) to (f): MR images acquired at a constant power of 0.08 W to demonstrate the increased SNR for LL3 (d) and LL4 (e) compared to the reference scan without Lenz lens (f).
(JPG)

S2 Fig SEM closeup of an electroplated LL4 lens. (JPG)

S3 Fig MRI reference sequence of an H_2_O phantom without Lenz lens. (JPG)

S4 Fig MRI sequence of an H_2_O phantom with LL1. (JPG)

S5 Fig MRI sequence of an H_2_O phantom with LL2. (JPG)

S6 Fig MRI sequence of an H_2_O phantom with LL3. (JPG)

S7 Fig MRI sequence of an H_2_O phantom with LL4. (JPG)
