# Supplementary figures and images for: Magnetic Lenz lenses improve the limit-of-detection in nuclear magnetic resonance
Source: PLoS One. 2017 Aug 15;12(8):e0182779. doi: 10.1371/journal.pone.0182779 (PMC5557590; doi:10.1371/journal.pone.0182779)

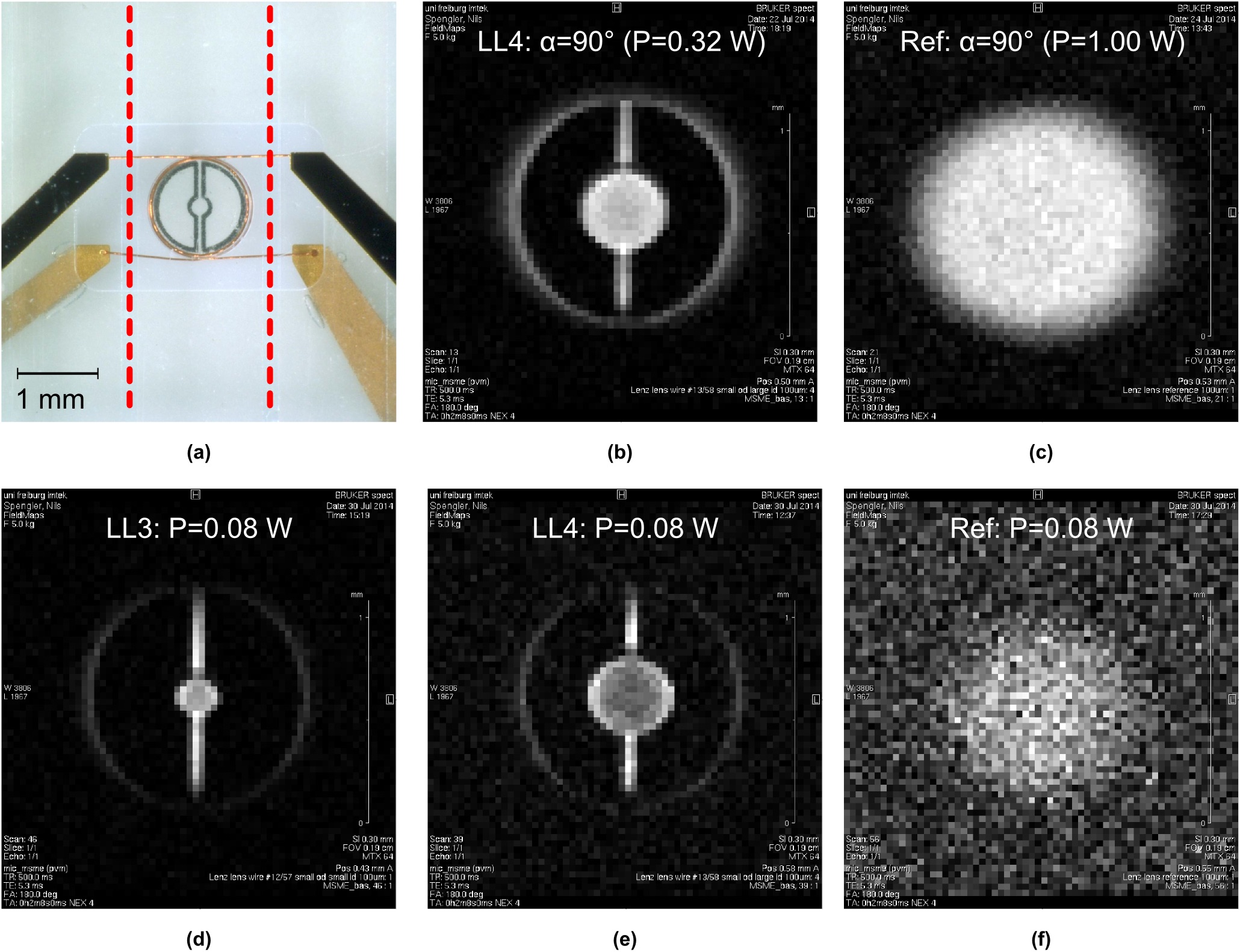

Supplement: S1 Fig — (a) Photograph of a LL3 chip inserted in between the 1.2 mm diameter micro Helmholtz coil pair used throughout the experiments. The microfluidic chamber filled with DI-water is depicted by broken red lines. (b) Acquired MR image using a LL4 chip with a 90° pulse at 0.32 W. (c) 90° reference scan without Lenz lens chip, which required a threefold higher power of 1.00 W. (d) to (f): MR images acquired at a constant power of 0.08 W to demonstrate the increased SNR for LL3 (d) and LL4 (e) compared to the reference scan without Lenz lens (f). (JPG) [file pone.0182779.s001.jpg]

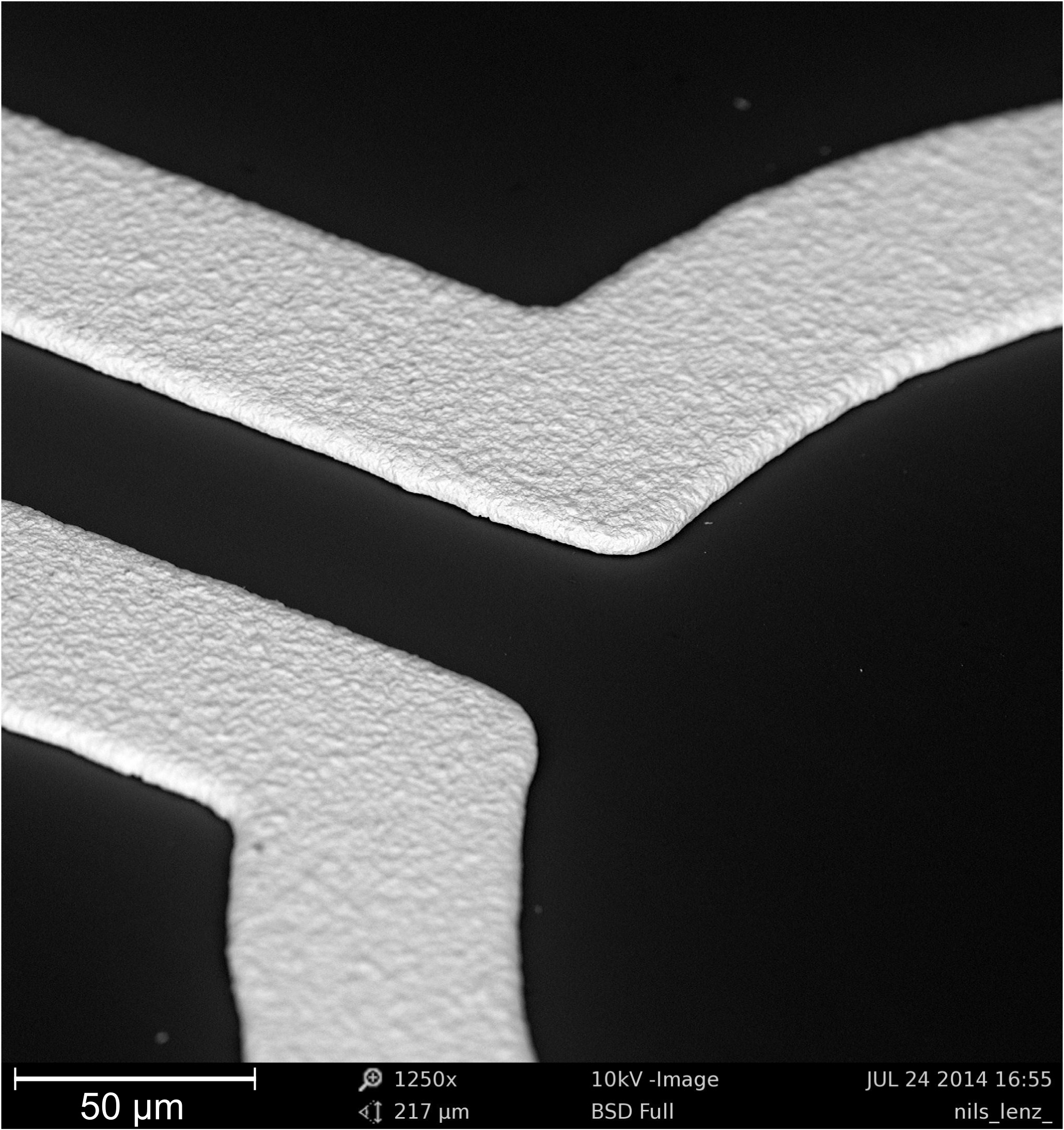

Supplement: S2 Fig — (JPG) [file pone.0182779.s002.jpg]

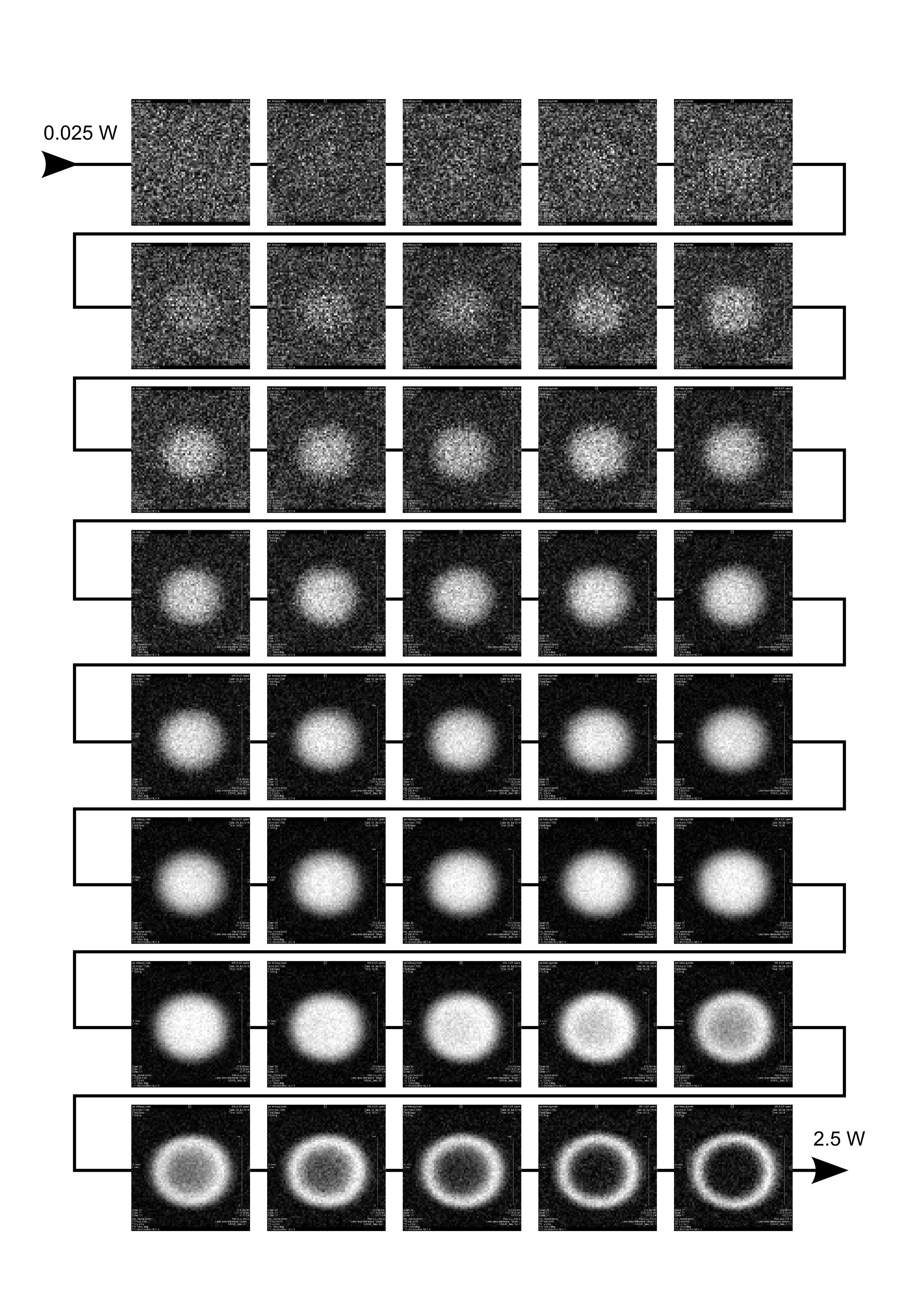

Supplement: S3 Fig — (JPG) [file pone.0182779.s003.jpg]

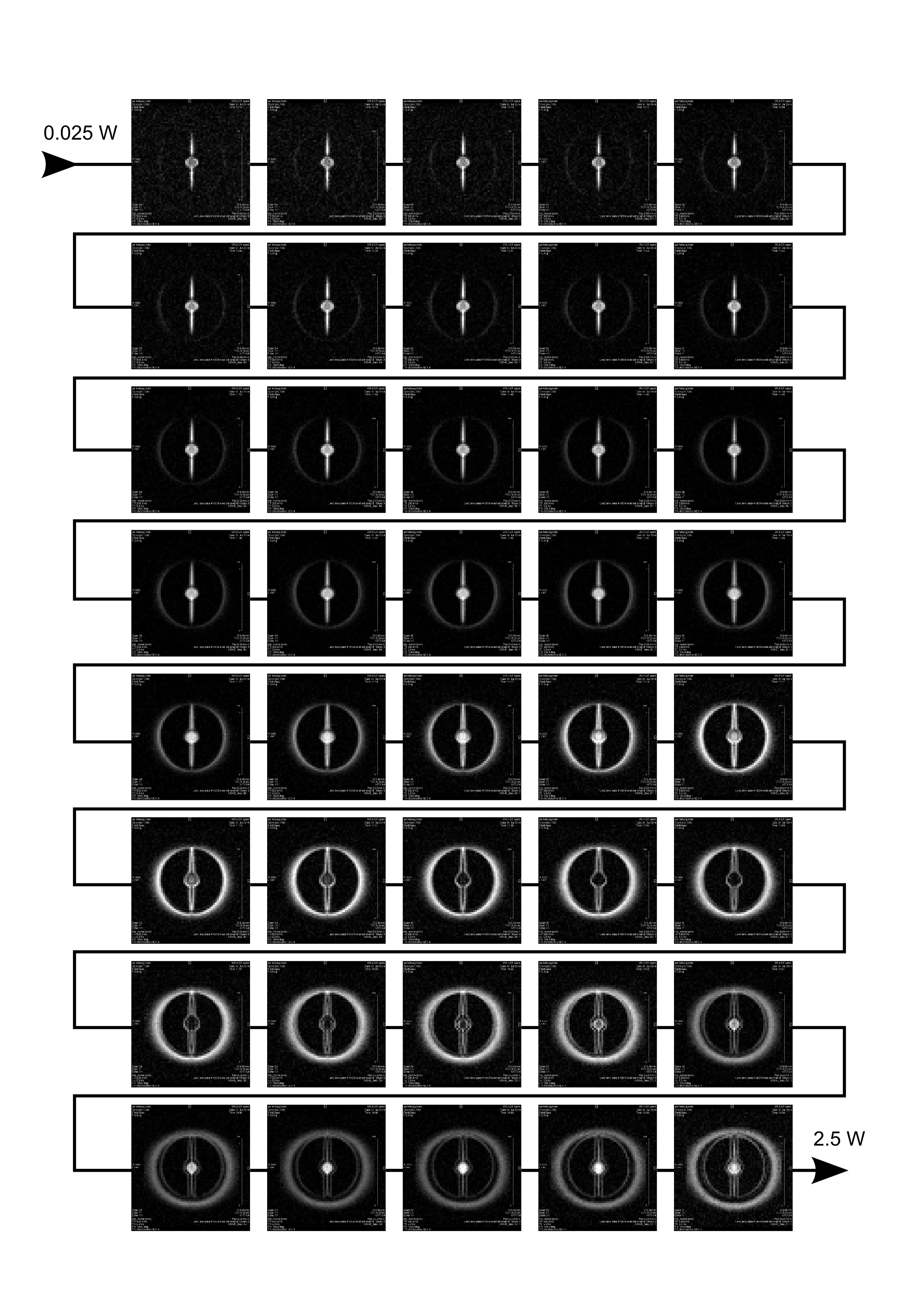

Supplement: S4 Fig — (JPG) [file pone.0182779.s004.jpg]

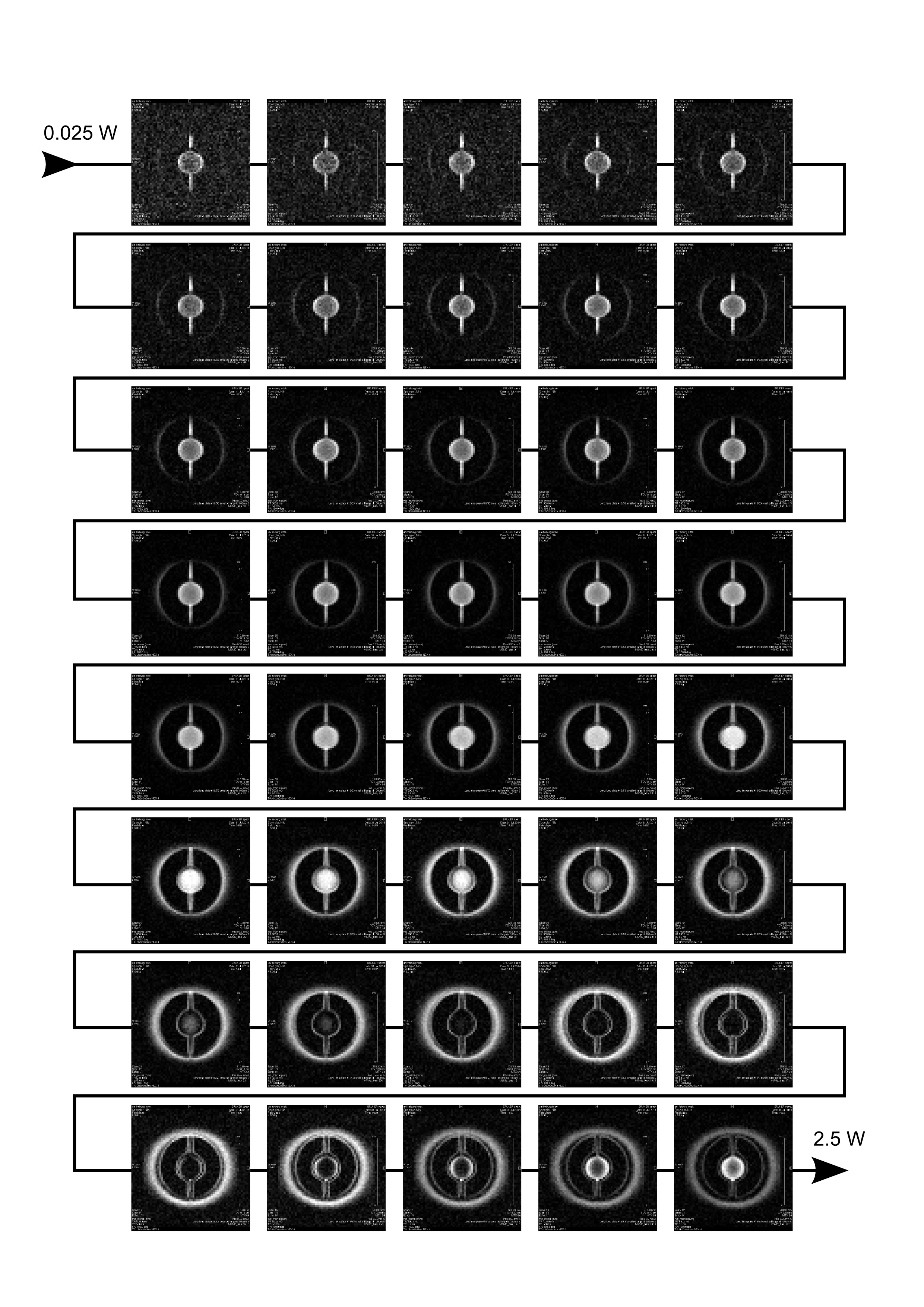

Supplement: S5 Fig — (JPG) [file pone.0182779.s005.jpg]

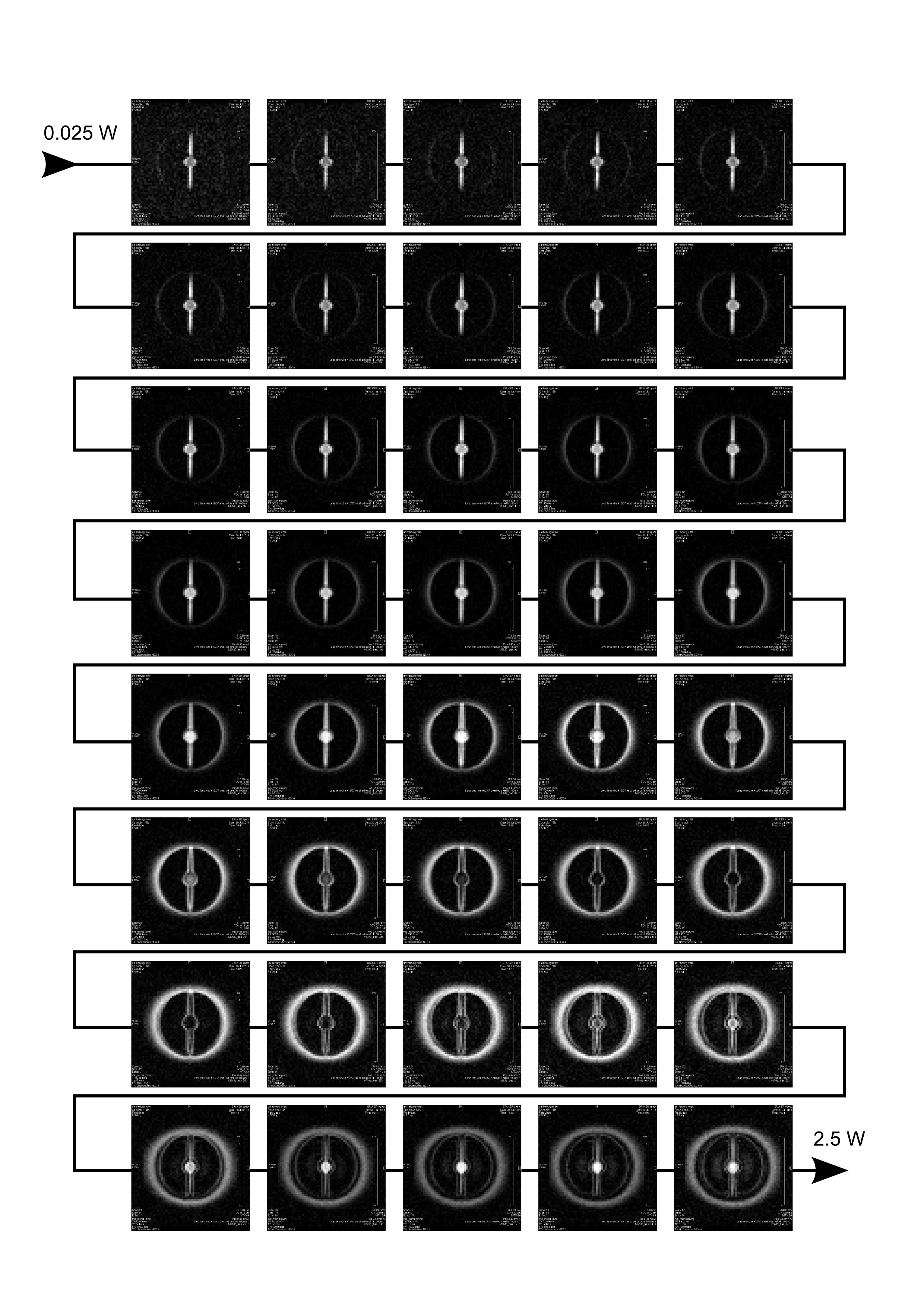

Supplement: S6 Fig — (JPG) [file pone.0182779.s006.jpg]

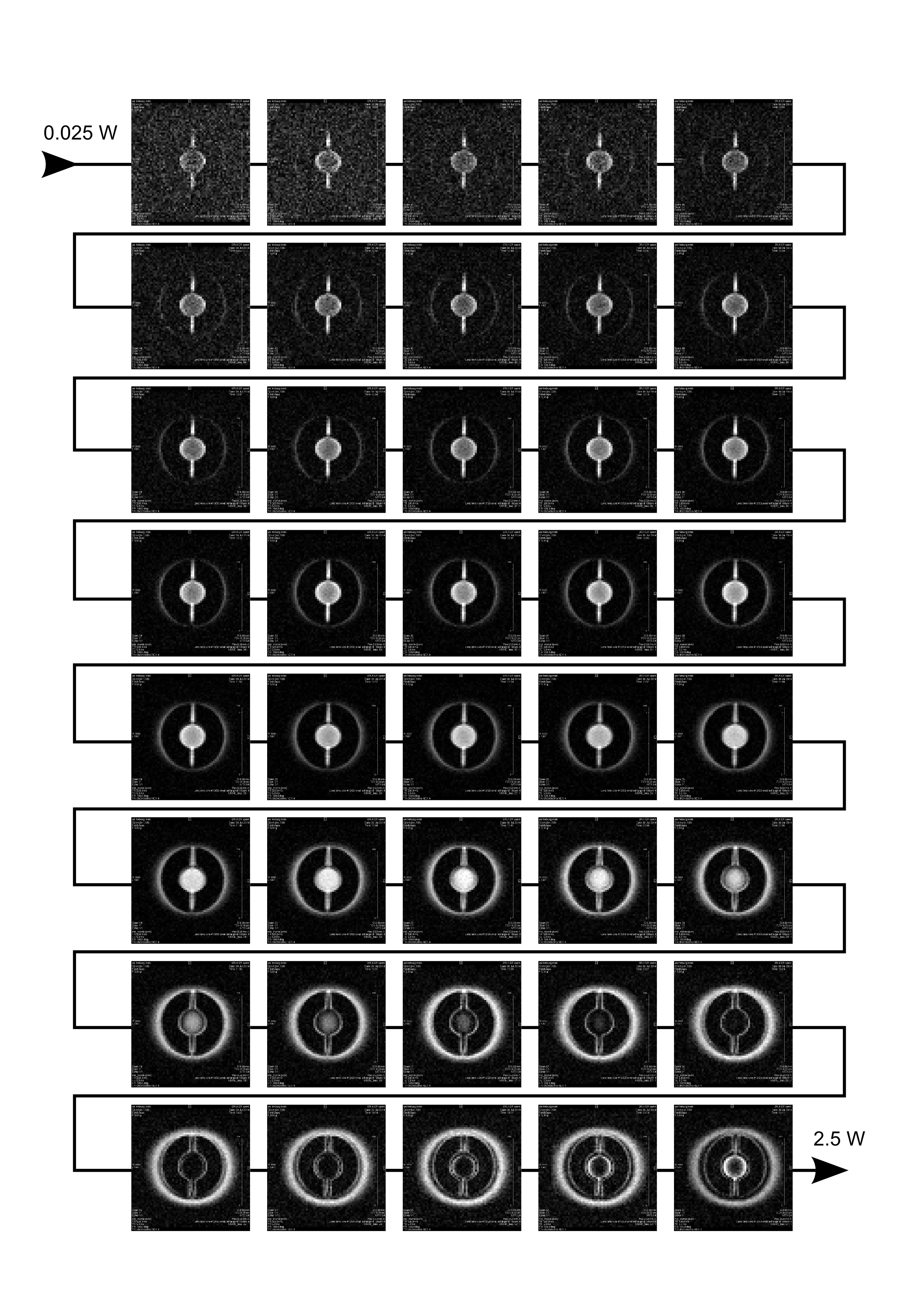

Supplement: S7 Fig — (JPG) [file pone.0182779.s007.jpg]
